# Human Endogenous Retrovirus K in the Crosstalk Between Cancer Cells Microenvironment and Plasticity: A New Perspective for Combination Therapy

**DOI:** 10.3389/fmicb.2018.01448

**Published:** 2018-07-02

**Authors:** Emanuela Balestrieri, Ayele Argaw-Denboba, Alessandra Gambacurta, Chiara Cipriani, Roberto Bei, Annalucia Serafino, Paola Sinibaldi-Vallebona, Claudia Matteucci

**Affiliations:** ^1^Department of Experimental Medicine and Surgery, University of Rome “Tor Vergata”, Rome, Italy; ^2^Department of Clinical Sciences and Translational Medicine, University of Rome “Tor Vergata”, Rome, Italy; ^3^Institute of Translational Pharmacology, National Research Council, Rome, Italy

**Keywords:** endogenous retroviruses, cancer plasticity, cancer therapy, cancer biomarker, combination therapy, reprogramming, stemness, tumor microenvironment

## Abstract

Abnormal activation of human endogenous retroviruses (HERVs) has been associated with several diseases such as cancer, autoimmunity, and neurological disorders. In particular, in cancer HERV activity and expression have been specifically associated with tumor aggressiveness and patient outcomes. Cancer cell aggressiveness is intimately linked to the acquisition of peculiar plasticity and heterogeneity based on cell stemness features, as well as on the crosstalk between cancer cells and the microenvironment. The latter is a driving factor in the acquisition of aggressive phenotypes, associated with metastasis and resistance to conventional cancer therapies. Remarkably, in different cell types and stages of development, HERV expression is mainly regulated by epigenetic mechanisms and is subjected to a very precise temporal and spatial regulation according to the surrounding microenvironment. Focusing on our research experience with HERV-K involvement in the aggressiveness and plasticity of melanoma cells, this perspective aims to highlight the role of HERV-K in the crosstalk between cancer cells and the tumor microenvironment. The implications for a combination therapy targeted at HERVs with standard approaches are discussed.

## Introduction

Human endogenous retroviruses are replication-defective proviruses comprising a portion of human genome (∼8%). HERVs are recognized as having a role in health maintenance (Vargas et al., 2009) and complex diseases (Suntsova et al., 2015; Meyer et al., 2017; Grandi and Tramontano, 2018) acting by remodeling structure and function of DNA. Although most of the HERV sequences have been inactivated over time, some of them remain active and, with LTRs, retain the genes encoding the structure, replication, and accessory proteins of retroviruses (Wildschutte et al., 2016). LTRs contain regulatory sequences and their activity essentially depends on the chromatin and CpG island methylation of their regulatory regions (Katoh and Kurata, 2013). In different cell types and stages of development, HERV expression is mainly regulated by epigenetic mechanisms and is subjected to regulation according to the surrounding microenvironment (Hurst and Magiorkinis, 2017). Numerous endogenous/exogenous factors lead to the activation of HERVs, including hormones (Buslei et al., 2015), cytokines (Manghera et al., 2016), cytotoxic chemicals/drugs (Diem et al., 2012; Mercorio et al., 2017), radiation (Schanab et al., 2011), vitamins (Liu et al., 2016), and interactions with microorganisms (Balada et al., 2009; Toufaily et al., 2011; Gonzalez-Hernandez et al., 2012).

In the last few decades, many studies have highlighted the involvement of HERVs in complex diseases, such as cancer, autoimmunity and neurological disorders (Young et al., 2013; Meyer et al., 2017). Although the research activity focused on the “omics” characterization of tumor from the primary site to the metastasis, the molecules that act as intermediaries between the epigenetic effect mediated by the microenvironment and cell fate haven’t been completely identified.

The ability of tumors to adapt to microenvironmental changes is embedded in their plasticity. Moreover, on the basis of the genetic predisposition, both differentiated and stem cells are driven toward transformation by the epigenetic pressure of the tumor niche and the microenvironmental changes (van den Hurk et al., 2012; Taddei et al., 2013).

An overview of the knowledge on HERVs related to tumors, in light of our experience in melanoma, is provided in order to achieve new insights into the contribution of HERV-K to the crosstalk between cancer cells and the tumor microenvironment. In addition, we suggest future perspectives on their potential therapeutic uses.

## Hervs in Cancer

Several mechanisms by which HERVs could produce pathological effects have been proposed, including generation of new variants of HERVs, insertional mutagenesis, and protein toxicity (Young et al., 2013). In this regard, HERV activation appears to influence the aggressiveness of different cancers, including seminoma, melanoma, leukemia, hepatocellular carcinoma, sarcoma, prostate, breast and colon cancer (Cegolon et al., 2013; Kassiotis, 2014; Pérot et al., 2015; Suntsova et al., 2015; Giebler et al., 2018). Likewise, the pathologic process of rheumatic disorders, systemic lupus erythematosus, multiple sclerosis, autism spectrum disorders, schizophrenia, bipolar disorder, psoriasis, type I diabetes, and systemic sclerosis shows a correlation with HERV activity (Alelú-Paz and Iturrieta-Zuazo, 2012; Balestrieri et al., 2012; Brodziak et al., 2012).

Several studies suggested that the aberrant activation of HERVs promotes tumorigenesis through oncogenic mechanisms, such as: (1) insertional mutagenesis with inactivation of tumor suppressor genes (Gerdes et al., 2016); (2) activation of downstream (proto-)oncogenes or genes involved in cell growth (Fan and Johnson, 2011); (3) expression of HERV-K oncogenes such as Rec and Np9 (Denne et al., 2007; Chen et al., 2013); (4) expression of HERV proteins involved in the fusion of tumor cells or immunosuppression (Downey et al., 2015); (5) disruption of cellular checkpoints (Kassiotis and Stoye, 2017; Lemaître et al., 2017).

Manifold HERV families have been identified; the HERV-K family is the most recently integrated in human genome, comprising 10 so-called HML subgroups (Subramanian et al., 2011). Of these, HML-2 subgroup maintains most of the ORFs actively transcribed. Due to differential transcript splicing and the deletion of 292-bp at the *pol* and *env* boundary, HML-2 produces the protein Env (single spliced) and accessory proteins Np9 and Rec (double spliced) (Armbruester et al., 2002; Büscher et al., 2006). Their identification helped to understand and characterize this subgroup of HERV-K in many tumors, including ovarian, breast and prostate cancer, melanoma, lymphomas, leukemias, and sarcomas (Cegolon et al., 2013; Kassiotis, 2014). HERV-K DNA-polymorphisms, mRNA and proteins have been detected in cancer cells; in addition, viral particles have been identified in tissue, serum, and cell lines (Hohn et al., 2013). Interestingly, HERV-K is involved in cell transformation and contributes to the metastatic phenotype (Downey et al., 2015). Accordingly, we demonstrated the reactivation of HERV-K under restrictive conditions to be strictly required in human melanoma cells to support the expansion of a subpopulation of cancer cells with stemness features (Serafino et al., 2009; Argaw-Denboba et al., 2017).

## Hervs and Stemness

Stem cells have self-renewal capacity and give rise to progeny capable of differentiating into diverse cell types. The transcription factors OCT4, SOX2, and NANOG have fundamental roles in maintaining the pluripotency and stemness features of hESCs and contribute to the reprogramming of adult somatic cells into iPSCs (Kashyap et al., 2009; Yamasaki et al., 2014). Recent studies showed HERV activity (mainly HERV-H and HERV-K) in hESCs and iPSC (Ohnuki et al., 2014; Grow et al., 2015). Specifically, LTR7/HERV-H is one of the transposable elements found more often at the binding sites of OCT4 and NANOG (Kunarso et al., 2010) and its targeting compromises the self-renewal functions (Wang et al., 2014). Furthermore, DNA hypomethylation at HERV-K LTRs elements together with transactivation by OCT4, increase HERV-K expression during embryogenesis. In addition, the overexpression of Rec in pluripotent cells increases the interferon-induced transmembrane protein 1 (IFITM1), suggesting a role of HERV-K in the immunoprotection of human embryos against viruses sensitive to the IFITM1-type restriction (Grow et al., 2015).

Possessing stemness features is crucial for cancer progression and metastasis. The generation of subpopulations with stemness features determines cancer self-renewal, proliferation and differentiation, allowing immune evasion and acquisition of resistance to therapy. These subpopulations, called CSCs, give rise to heterogeneous cell populations and maintain an undifferentiated state that equips them with the plasticity required to survive environmental stress (Aponte and Caicedo, 2017; Ramos et al., 2017).

The role of HERVs in stemness and the acquisition of cancer stemness are linked by a complex crosstalk of cellular signals, in which microenvironmental changes play a significant role (Cabrera et al., 2015; Argaw-Denboba et al., 2017; Flavahan et al., 2017).

## The Role of HERV-K in the Plasticity of Cancer Cells: Our Point of View in Melanoma

Several studies suggest that the tumorigenesis is determined by genetic alterations, which contribute to transformation, as well as by external factors present in the cancer microenvironment. The microenvironment is therefore considered a part of the tumor that constantly changes in parallel with cancer progression, as a result of bidirectional interactions between tumor cells and cellular and molecular components of their “niche” (Plaks et al., 2015; Wang et al., 2017). These interactions are essential for the establishment of a permissive stem cell microenvironment, providing a fine balance between self-renewal/differentiation and quiescence/proliferation. The tumor microenvironment is characterized by adverse growth conditions (hypoxia and acidosis), which trigger a stress response in cancer cells that, with molecules such as cytokines and growth factors, is instrumental in phenotype switching, angiogenesis, tumor growth, and immune evasion.

Cellular plasticity is fundamental for tumor progression and metastasis, to adapt to changes in the microenvironment (Taddei et al., 2013). Aggressive cancer cells share many characteristics with embryonal progenitors, expressing developmental genes that allow the differentiation into a wide range of cell lineages, including neural, mesenchymal, and endothelial cells. This mimicry of other cell lineages becomes essential in the cancer’s ability to adapt to microenvironmental changes. For instance, melanoma cells show phenotypic heterogeneity and maintain their morphological and biological plasticity despite repeated cloning (Bröcker et al., 1991; Hendrix et al., 2003; Boiko et al., 2010).

Several studies from our and other groups demonstrated that HERV-K (HML-2), has a potential aggravating role in malignant melanoma and in immune escape during metastasis (Büscher et al., 2006; Serafino et al., 2009; Argaw-Denboba et al., 2017). Since HERV-K is responsive to microenvironmental changes, and melanoma cells are strongly associated with epigenetic and microenvironmental anomalies, the association of HERV-K activation with carcinogenesis is particularly intriguing (Li et al., 2015; Roesch, 2015).

**FIGURE 1:**
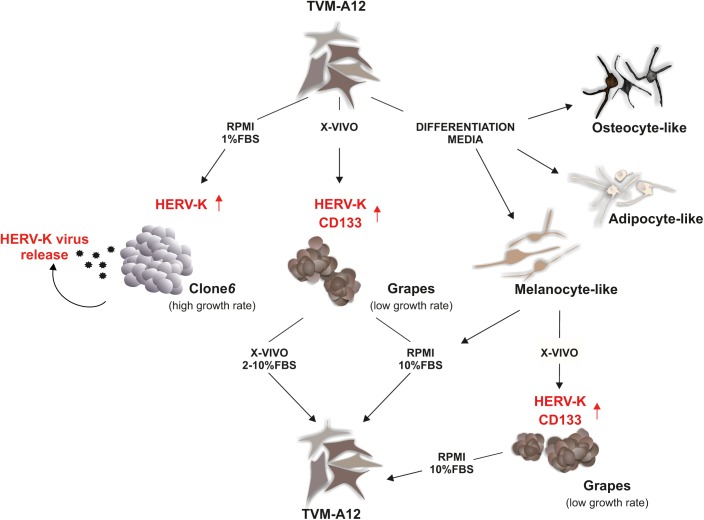
HERV-K as the master of melanoma plasticity in tumor adaptation to microenvironment. In response to different types of media, TVM-A12 melanoma cells change morphology and functional properties. The acquisition of undifferentiated and of stemness features under microenvironment alterations is HERV-K-dependent and related to increased malignancy, metastatic potential, and immune evasion.

Our group has established and characterized a metastatic human melanoma cell line, termed TVM-A12, that is highly heterogeneous, plastic and responds strongly to microenvironmental alterations (Melino et al., 1993; Serafino et al., 2009; Argaw-Denboba et al., 2017) (**Figure 1**). This has supplied a model to study the crosstalk between HERVs and cancer cells in a changing microenvironment. The TVM-A12 cellular monolayer is characterized by the presence of cells with different morphologies including small ovoid, spindle polygonal and large dendritic forms. Notably, the multiple morphology with melanin production persisted after years of continued passage in culture. When grown in different media, despite changing the morphology and functional characteristics, TVM-A12 retain the ability to restore the original phenotype if standard conditions are re-established. Peculiarly, when cultured in specific media that promote differentiation, TVM-A12 cells show specific phenotypes of melanogenic, adipogenic, and osteogenic lineages (unpublished data) (**Figure 1**). This morphological transition correlated with the change of culture media, without committing to terminal differentiation, has been previously described and is considered a hallmark of stemness in melanoma cells (Zhu et al., 2014).

TVM-A12 cells were also cultured at low serum concentrations (RPMI with 1%FBS), a recognized protocol for inducing microenvironmental stress conditions *in vitro*. This prompted a change in their phenotype, switching from adherent to suspension cells, generating a highly proliferating cell line called Clone6 (**Figure 1**). A major event for the generation of metastatic tumor cells is the inhibition of anoikis, the programmed cell death pathway induced by loss of integrin-mediated cell matrix interactions, revealed *in vitro* by the ability of cells to grow in an anchorage-independent manner (Paoli et al., 2013). In the case of Clone6, following cell detachment, cells undergo uncontrolled growth and show loss of expression of immune recognition molecules (MHC-I, Melan A/MART-1), loss of melanin production and ability to generate tumor masses in mice (unpublished data), as one would expect from highly malignant cells. Uniquely, Clone6 cells are unable to return to the original phenotype when standard culture conditions are re-established, and there is a marked transcriptional activation of HERV-K with the concomitant production and release of viral particles, along with these phenotypic and functional changes. The generation of Clone6 from TVM-A12 is shown to be dependent on HERV-K as down-regulation by RNA interference prevents it.

When switching to a serum free medium, such as X-VIVO (typically used for stem cells), TVM-A12 generated non-adherent dark cellular aggregates called Grapes, with a low growth rate (**Figure 1**). Grapes are characterized by increased expression of CSCs markers (CD133 and nestin), loss of expression of immune recognition molecules (MHC-I, Melan A/MART-1) and an increase in melanoma progression and metastasis associated markers (CD10 and CXCR4). This phenotype switch is accompanied by an increased expression of HERV-K, and the link to microenvironmental changes is confirmed by a strong down-regulation of HERV-K expression when cells are returned to media containing serum (RPMI with 10%FBS; X-VIVO with 2–10%FBS). Once again confirming its important role, the silencing of HERV-K in TVM-A12 leads to a reduction in Grapes formation and an induction of cell death.

This HERV-K interference, during Grapes generation, also specifically inhibits the expansion of a CD133+ subpopulation with stemness features, demonstrating the requirement of HERV-K activation to sustain the expansion of this subpopulation. Microenvironmental stress is indicated as critical for regulating stemness of tumor cells (Plaks et al., 2015). This subpopulation is characterized by recognized hallmarks of cancer aggressiveness such as high expression of OCT4, self-renewing, migration and invasion capacity. Remarkably, when these cells are treated with NNRTIs such as efavirenz and nevirapine, the results mimic HERV-K interference, with a decrease in HERV-K expression and a concomitant cell death induction. We cannot be certain if the effect of NNRTIs treatment is due to a direct inhibition of the reverse transcriptase of HERV-K or is mediated by the action on other cellular components such as LINE-1 (Sinibaldi-Vallebona et al., 2011).

Tumors are graded and evaluated based on their degree of cellular differentiation, more malignant cancers lose the characteristics of the original tissue and approach a more stem-cell-like state (Lathia and Liu, 2017). Given this, it appears that HERV-K inhibition offers a promising avenue of research for combination therapy in cancer.

## HERV-K and the Acquisition of Cancer Hallmarks: Rationale for Combination Therapies

**FIGURE 2:**
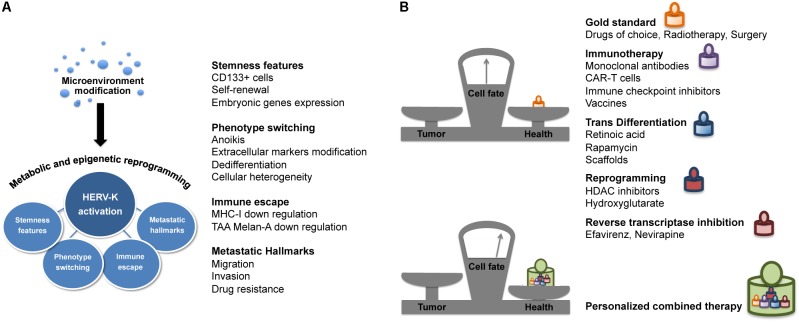
HERV-K provides new avenues for combination therapy. **(A)** HERV-K and cancer hallmarks. In melanoma cells, microenvironment modifications lead to the increase of HERV-K transcriptional activity associated to cell plasticity and different hallmarks of cancer such as phenotype switching, stemness features, immune evasion, and metastatic properties. **(B)** HERV-K targeting in cancer combination therapy. Targeting HERV-K in association with conventional and innovative therapies such as immunotherapy, cell transdifferentiation/reprogramming-inducing agents, epigenetic modifiers and antiretrovirals, could shift the balance in the search for effective and less toxic cancer treatment.

Cell heterogeneity and plasticity are the main drivers of the clonal evolution of genetic resistance and the emergence of highly metastatic tumor phenotypes resistant to conventional chemotherapies and radiation (Skvortsov et al., 2014; Roesch, 2015). The phenotype-switching ability of melanoma cells in response to the microenvironment drives the dynamicity of its immune escape and malignant characteristics (Li et al., 2015). Accordingly, our studies on melanoma have shown how the activation of HERV-K under microenvironmental stress induces and maintains tumor cell plasticity and determines the acquisition of the typical cancer hallmarks, such as changes in phenotype, stemness feature, immune evasion, and metastasis (**Figure 2A**). Cancer progression is accompanied by metabolic alterations and epigenetic reprogramming. The tumor microenvironment, poor in nutrients and oxygen, is responsible for the metabolic switch observed in cancer cells. The accumulation of glycolysis metabolic products, such as lactate, induces local immune suppression, which facilitates tumor progression and metastasis (Renner et al., 2017). Both cellular nutrient metabolism and chromatin organization are remodeled in cancer cells, and these alterations play a key role in tumor development and growth. Indeed, many chromatin modifying-enzymes utilize metabolic intermediates as cofactors or substrates, and recent studies have shown that the epigenome is sensitive to cellular metabolism (Yun et al., 2012). Thus, while epigenetic alterations can modify the expression of metabolic enzymes, the metabolic reprogramming can affect the cancer cell epigenome as well (by DNA methylation and histone modifications). One of the most important events for the development and progression of cancer is global DNA hypomethylation (Ehrlich, 2009; Sandoval and Esteller, 2012); indeed, the expression of HERV-K is strongly associated with hypomethylation (Stengel et al., 2010; Kreimer et al., 2013), and with increased genomic instability and transcriptome activity (Romanish et al., 2010). In this context, the identification and study of mechanisms and regulators of the metabolic switch and epigenetic modifiers, with an eye toward targeted therapies to be used in combination, should provide more effective cancer therapies.

The eradication of tumors and prevention of recurrences are current challenges in cancer therapy. Thus, starting from conventional cancer treatments, including chemotherapy, radiotherapy and surgery, new combination approaches are needed. In this view, we consider HERV-K targeting as a strategy to improve response to therapy (**Figure 2B**). The enhancement of patient’s immune response plays a key role in the treatment of cancer. Indeed, in the field of cancer immunotherapy, progress has been made in the development of new technologies aimed at boosting the immune system, such as monoclonal antibodies and engineered CAR-T against tumor antigens (Khalil et al., 2016). Actually, targeting of the HERV-K envelope protein by CAR-T cells has already been reported as a potential immunotherapeutic approach for melanoma and other tumors (Krishnamurthy et al., 2015). Targeted immunotherapy research demonstrated the potential of anti-HML-2-Env antibodies in inhibiting tumor growth and inducing apoptosis, both *in vitro* and *in vivo* mouse models (Wang-Johanning et al., 2012). Moreover, based on the immunogenic property of HERV-K proteins (Reis et al., 2013), studies are underway on a peptide-based vaccine derived from HERV-K in order to control the spread of cancer (Kraus et al., 2013).

Another approach is the identification of the microenvironmental factors and the corresponding signal transduction pathways that are responsible for transdifferentiation of cancer cells. These could be potential targets for a new therapeutic approach for cancer reprogramming into a differentiated state, with a decrease or even loss of cancer stemness features and malignancy (Carpentieri et al., 2016). One promising result in this direction has been the achievement of a new type of cancer cell reprogramming: the osteogenic differentiation of neuroblastoma cells, switched to a different germ layer through rapamycin induction in the presence of a scaffold, without an intermediate iPSCs step (Carpentieri et al., 2015). It would be of interest to study how the expression of retroelements is regulated during the cancer transdifferentiation process.

Histone deacetylate inhibitors are currently used in the clinical setting as anticancer agents that alter the regulation of histone proteins. HDACi can modify the acetylation status of histones, resulting in the induction of cell cycle arrest, apoptosis or differentiation (Eckschlager et al., 2017). HDACi potentially re-activate HERVs, however, the beneficial or detrimental effects of epigenetic drugs on HERV modulation are currently discussed (Chiappinelli et al., 2015; Hurst et al., 2016; Daskalakis et al., 2018; White et al., 2018).

In a broader context, our group and other authors had already suggested intervening on the activity of retroelements with antiretroviral drugs (Sinibaldi-Vallebona et al., 2011; Argaw-Denboba et al., 2017; Contreras-Galindo et al., 2017), which now more than ever appears as a promising new component in future combination therapies in cancer.

## Future Directions

In this scenario, the responsiveness to external stimuli of HERVs attributes to these genetic elements a high relevance in the crosstalk between tumor and microenvironment. Based on our experience we have demonstrated that HERV-K is fundamental in the acquisition of stemness features and aggressiveness under the pressure of the microenvironment. Following the path indicated by our results on melanoma cells, we aim to widen the scope and depth of our knowledge of HERV-K in cancer plasticity, by exploring other cancer types and by deciphering the molecular pathways underlying the responsiveness of HERV-K to the microenvironment. We believe that future combination therapies able to target HERV-K and other retroelements will become indispensable weapons in a wider arsenal for fighting cancers. Therefore, we propose that expanding the range of therapeutic options will allow defining personalized combination therapies in the future.

## Author Contributions

All authors listed have made substantial, direct and intellectual contributions to this perspective, revised and approved the final version of the manuscript for publication.

## Conflict of Interest Statement

The authors declare that the research was conducted in the absence of any commercial or financial relationships that could be construed as a potential conflict of interest.

## Abbreviations

CAR-T: chimeric antigen receptor T cells
Clone6: highly proliferating melanoma cell line
CSCs: cancer stem cells
FBS: fetal bovine serum
Grapes: non-adherent dark cellular aggregates
HDACi: histone deacetylate inhibitors
HERVs: human endogenous retroviruses
hESCs: human embryonic stem cells
HML: human endogenous MMTV-like
IFITM1: interferon induced transmembrane protein 1
iPSCs: induced pluripotent stem cells
LTRs: long terminal repeats
Melan A/MART-1: protein melanoma-A/melanoma antigen recognized by T cells 1
MHC-I: major histocompatibility complex
NANOG: DNA binding homeobox transcription factor
NNRTIs: non-nucleoside reverse-transcriptase inhibitors
OCT4: octamer-binding transcription factor 4
ORFs: open reading frames
RPMI: standard medium
SOX2: transcription factor, Sex determining region Y-box 2
TVM-A12: human melanoma cell line
X-VIVO: serum free medium

## References

[B1] Alelú-Paz R. Iturrieta-Zuazo I. (2012). Human endogenous retroviruses: their possible role in the molecular etiology of the schizophrenia. *Open J. Gen.* 2 70–76. 10.4236/ojgen.2012.21009 22067224

[B2] Aponte P. M. Caicedo A. (2017). Stemness in cancer: stem cells, cancer stem cells, and their microenvironment. *Stem Cells Int.* 2017 5619472. 10.1155/2017/5619472 28473858 PMC5394399

[B3] Argaw-Denboba A. Balestrieri E. Serafino A. Cipriani C. Bucci I. Sorrentino R. (2017). HERV-K activation is strictly required to sustain CD133+ melanoma cells with stemness features. *J. Exp. Clin. Cancer Res.* 36:20. 10.1186/s13046-016-0485-x 28125999 PMC5270369

[B4] Armbruester V. Sauter M. Krautkraemer E. Meese E. Kleiman A. Best B. (2002). A novel gene from the human endogenous retrovirus K expressed in transformed cells. *Clin. Cancer Res.* 8 1800–1807. 12060620

[B5] Balada E. Ordi-Ros J. Vilardell-Tarrés M. (2009). Molecular mechanisms mediated by human endogenous retroviruses (HERVs) in autoimmunity. *Rev. Med. Virol.* 19 273–286. 10.1002/rmv.622 19714703

[B6] Balestrieri E. Arpino C. Matteucci C. Sorrentino R. Pica F. Alessandrelli R. (2012). HERVs expression in autism spectrum disorders. *PLoS One* 7:e48831. 10.1371/journal.pone.0048831 23155411 PMC3498248

[B7] Boiko A. D. Razorenova O. V. van de Rijn M. Swetter S. M. Johnson D. L. Ly D. P. (2010). Human melanoma-initiating cells express neural crest nerve growth factor receptor CD271. *Nature* 466 133–137. 10.1038/nature09161 20596026 PMC2898751

[B8] Bröcker E. B. Magiera H. Herlyn M. (1991). Nerve growth and expression of receptors for nerve growth factor in tumors of melanocyte origin. *J. Invest. Dermatol.* 96 662–665. 10.1111/1523-1747.ep12470585 1850772

[B9] Brodziak A. Ziółko E. Muc-Wierzgoń M. Nowakowska-Zajdel E. Kokot T. Klakla K. (2012). The role of human endogenous retroviruses in the pathogenesis of autoimmune diseases. *Med. Sci. Monit.* 18 RA80–RA88. 10.12659/MSM.882892 22648263 PMC3560723

[B10] Büscher K. Hahn S. Hofmann M. Trefzer U. Ozel M. Sterry W. (2006). Expression of the human endogenous retrovirus-K transmembrane envelope, Rec and Np9 proteins in melanomas and melanoma cell lines. *Melanoma Res.* 16 223–234. 10.1097/01.cmr.0000215031.07941.ca 16718269

[B11] Buslei R. Strissel P. L. Henke C. Schey R. Lang N. Ruebner M. (2015). Activation and regulation of endogenous retroviral genes in the human pituitary gland and related endocrine tumours. *Neuropathol. Appl. Neurobiol.* 41 180–200. 10.1111/nan.12136 24635849

[B12] Cabrera M. C. Hollingsworth R. E. Hurt E. M. (2015). Cancer stem cell plasticity and tumor hierarchy. *World J. Stem Cells* 7 27–36. 10.4252/wjsc.v7.i1.27 25621103 PMC4300934

[B13] Carpentieri A. Cozzoli E. Scimeca M. Bonanno E. Sardanelli A. M. Gambacurta A. (2015). Differentiation of human neuroblastoma cells toward the osteogenic lineage by mTOR inhibitor. *Cell Death Dis.* 6 e1974. 10.1038/cddis.2015.244 26561783 PMC4670915

[B14] Carpentieri A. Diedenhofen G. Gambacurta G. (2016). Back on track: new perspectives on cancer cell reprogramming. *Single Cell Biol.* 5:151 10.4172/2168-9431.1000151

[B15] Cegolon L. Salata C. Weiderpass E. Vineis P. Palù G. Mastrangelo G. (2013). Human endogenous retroviruses and cancer prevention: evidence and prospects. *BMC Cancer* 13:4. 10.1186/1471-2407-13-4 23282240 PMC3557136

[B16] Chen T. Meng Z. Gan Y. Wang X. Xu F. Gu Y. (2013). The viral oncogene Np9 acts as a critical molecular switch for co-activating β-catenin, ERK, Akt and Notch1 and promoting the growth of human leukemia stem/progenitor cells. *Leukemia* 27 1469–1478. 10.1038/leu.2013.8 23307033

[B17] Chiappinelli K. B. Strissel P. L. Desrichard A. Li H. Henke C. Akman B. (2015). Inhibiting DNA methylation causes an interferon response in cancer via dsRNA including endogenous retroviruses. *Cell* 162 974–986. 10.1016/j.cell.2015.07.011 26317466 PMC4556003

[B18] Contreras-Galindo R. Dube D. Fujinaga K. Kaplan M. H. Markovitz D. M. (2017). Susceptibility of human endogenous retrovirus type K to reverse transcriptase inhibitors. *J. Virol.* 91 e1309–e1317. 10.1128/JVI.01309-17 28931682 PMC5686744

[B19] Daskalakis M. Brocks D. Sheng Y. H. Islam M. S. Ressnerova A. Assenov Y. (2018). Reactivation of endogenous retroviral elements via treatment with DNMT- and HDAC-inhibitors. *Cell Cycle* 10.1080/15384101.2018.1442623 [Epub ahead of print]. 29633898 PMC6056222

[B20] Denne M. Sauter M. Armbruester V. Licht J. D. Roemer K. Mueller-Lantzsch N. (2007). Physical and functional interactions of human endogenous retrovirus proteins Np9 and rec with the promyelocytic leukemia zinc finger protein. *J. Virol.* 81 5607–5616. 10.1128/JVI.02771-06 17360752 PMC1900259

[B21] Diem O. Schäffner M. Seifarth W. Leib-Mösch C. (2012). Influence of antipsychotic drugs on human endogenous retrovirus (HERV) transcription in brain cells. *PLoS One* 7:e30054. 10.1371/journal.pone.0030054 22253875 PMC3256206

[B22] Downey R. F. Sullivan F. J. Wang-Johanning F. Ambs S. Giles F. J. Glynn S. A. (2015). Human endogenous retrovirus K and cancer: Innocent bystander or tumorigenic accomplice? *Int. J. Cancer* 137 1249–1257. 10.1002/ijc.29003 24890612 PMC6264888

[B23] Eckschlager T. Plch J. Stiborova M. Hrabeta J. (2017). Histone deacetylase inhibitors as anticancer drugs. *Int. J. Mol. Sci.* 18:E1414. 10.3390/ijms18071414 28671573 PMC5535906

[B24] Ehrlich M. (2009). DNA hypomethylation in cancer cells. *Epigenomics* 1 239–259. 10.2217/epi.09.33 20495664 PMC2873040

[B25] Fan H. Johnson C. (2011). Insertional oncogenesis by non-acute retroviruses: implications for gene therapy. *Viruses* 3 398–422. 10.3390/v3040398 21994739 PMC3186009

[B26] Flavahan W. A. Gaskell E. Bernstein B. E. (2017). Epigenetic plasticity and the hallmarks of cancer. *Science* 357:eaal2380. 10.1126/science.aal2380 28729483 PMC5940341

[B27] Gerdes P. Richardson S. R. Mager D. L. Faulkner G. J. (2016). Transposable elements in the mammalian embryo: pioneers surviving through stealth and service. *Genome Biol.* 17:100. 10.1186/s13059-016-0965-5 27161170 PMC4862087

[B28] Giebler M. Staege M. S. Blauschmidt S. Ohm L. I. Kraus M. Würl P. (2018). Elevated HERV-K expression in soft tissue sarcoma is associated with worsened relapse-free survival. *Front. Microbiol.* 9:211. 10.3389/fmicb.2018.00211 29487589 PMC5816752

[B29] Gonzalez-Hernandez M. J. Swanson M. D. Contreras-Galindo R. Cookinham S. King S. R. Noel R. J. Jr. (2012). Expression of human endogenous retrovirus type K (HML-2) is activated by the Tat protein of HIV-1. *J. Virol.* 86 7790–7805. 10.1128/JVI.07215-11 22593154 PMC3421662

[B30] Grandi N. Tramontano E. (2018). HERV envelope proteins: physiological role and pathogenic potential in cancer and autoimmunity. *Front. Microbiol.* 9:462. 10.3389/fmicb.2018.00462 29593697 PMC5861771

[B31] Grow E. J. Flynn R. A. Chavez S. L. Bayless N. L. Wossidlo M. Wesche D. J. (2015). Intrinsic retroviral reactivation in human preimplantation embryos and pluripotent cells. *Nature* 522 221–225. 10.1038/nature14308 25896322 PMC4503379

[B32] Hendrix M. J. Seftor E. A. Hess A. R. Seftor R. E. (2003). Molecular plasticity of human melanoma cells. *Oncogene* 22 3070–3075. 10.1038/sj.onc.1206447 12789282

[B33] Hohn O. Hanke K. Bannert N. (2013). HERV-K(HML-2), the best preserved family of HERVs: endogenization, expression, and implications in health and disease. *Front. Oncol.* 3:246. 10.3389/fonc.2013.00246 24066280 PMC3778440

[B34] Hurst T. Pace M. Katzourakis A. Phillips R. Klenerman P. Frater J. (2016). Human endogenous retrovirus (HERV) expression is not induced by treatment with the histone deacetylase (HDAC) inhibitors in cellular models of HIV-1 latency. *Retrovirology* 13:10. 10.1186/s12977-016-0242-4 26852322 PMC4744380

[B35] Hurst T. P. Magiorkinis G. (2017). Epigenetic control of human endogenous retrovirus expression: focus on regulation of long-terminal repeats (LTRs). *Viruses* 9:E130. 10.3390/v9060130 28561791 PMC5490807

[B36] Kashyap V. Rezende N. C. Scotland K. B. Shaffer S. M. Persson J. L. Gudas L. J. (2009). Regulation of stem cell pluripotency and differentiation involves a mutual regulatory circuit of the NANOG, OCT4, and SOX2 pluripotency transcription factors with polycomb repressive complexes and stem cell microRNAs. *Stem Cells Dev.* 18 1093–1108. 10.1089/scd.2009.0113 19480567 PMC3135180

[B37] Kassiotis G. (2014). Endogenous retroviruses and the development of cancer. *J. Immunol.* 192 1343–1349. 10.4049/jimmunol.1302972 24511094 PMC3925786

[B38] Kassiotis G. Stoye J. P. (2017). Making a virtue of necessity: the pleiotropic role of human endogenous retroviruses in cancer. *Philos. Trans. R. Soc. Lond. B Biol. Sci.* 372:20160277. 10.1098/rstb.2016.0277 28893944 PMC5597744

[B39] Katoh I. Kurata S. (2013). Association of endogenous retroviruses and long terminal repeats with human disorders. *Front. Oncol.* 3:234. 10.3389/fonc.2013.00234 24062987 PMC3769647

[B40] Khalil D. N. Smith E. L. Brentjens R. J. Wolchok J. D. (2016). The future of cancer treatment: immunomodulation, CARs and combination immunotherapy. *Nat. Rev. Clin. Oncol.* 13 273–290. 10.1038/nrclinonc.2016.25 26977780 PMC5551685

[B41] Kraus B. Fischer K. Büchner S. M. Wels W. S. Löwer R. Sliva K. (2013). Vaccination directed against the human endogenous retrovirus-K envelope protein inhibits tumor growth in a murine model system. *PLoS One* 8:e72756. 10.1371/journal.pone.0072756 24023643 PMC3758348

[B42] Kreimer U. Schulz W. A. Koch A. Niegisch G. Goering W. (2013). HERV-K and LINE-1 DNA methylation and reexpression in urothelial carcinoma. *Front. Oncol.* 2013:255. 10.3389/fonc.2013.00255 24133654 PMC3783855

[B43] Krishnamurthy J. Rabinovich B. A. Mi T. Switzer K. C. Olivares S. Maiti S. N. (2015). Genetic engineering of T cells to target HERV-K, an ancient retrovirus on melanoma. *Clin. Cancer Res.* 21 3241–3251. 10.1158/1078-0432.CCR-14-3197 25829402 PMC4506228

[B44] Kunarso G. Chia N. Y. Jeyakani J. Hwang C. Lu X. Chan Y. S. (2010). Transposable elements have rewired the core regulatory network of human embryonic stem cells. *Nat. Genet.* 42 631–634. 10.1038/ng.600 20526341

[B45] Lathia J. D. Liu H. (2017). Overview of cancer stem cells and stemness for community oncologists. *Target. Oncol.* 12 387–399. 10.1007/s11523-017-0508-3 28664387 PMC5524873

[B46] Lemaître C. Tsang J. Bireau C. Heidmann T. Dewannieux M. (2017). A human endogenous retrovirus-derived gene that can contribute to oncogenesis by activating the ERK pathway and inducing migration and invasion. *PLoS Pathog.* 13:e1006451. 10.1371/journal.ppat.1006451 28651004 PMC5501692

[B47] Li F. Z. Dhillon A. S. Anderson R. L. McArthur G. Ferrao P. T. (2015). Phenotype switching in melanoma: implications for progression and therapy. *Front. Oncol.* 5:31. 10.3389/fonc.2015.00031 25763355 PMC4327420

[B48] Liu M. Ohtani H. Zhou W. Ørskov A. D. Charlet J. Zhang Y. W. (2016). Vitamin C increases viral mimicry induced by 5-aza-2’-deoxycytidine. *Proc. Natl. Acad. Sci. U.S.A.* 113 10238–10244. 10.1073/pnas.1612262113 27573823 PMC5027469

[B49] Manghera M. Ferguson-Parry J. Lin R. Douville R. N. (2016). NF-κB and IRF1 induce endogenous retrovirus K expression via interferon-stimulated response elements in its 5’ long terminal repeat. *J. Virol.* 90 9338–9349. 10.1128/JVI.01503-16 27512062 PMC5044829

[B50] Melino G. Sinibaldi-Vallebona P. D’Atri S. Annicchiarico-Petruzzelli M. Rasi G. Catani M. V. (1993). Characterization of three melanoma cell lines (TVM-A12, TVM-A-197, TVM-BO): sensitivity to lysis and effect of retinoic acid. *Clin. Chem. Enzymol. Commun.* 6 105–119.

[B51] Mercorio R. Bonzini M. Angelici L. Iodice S. Delbue S. Mariani J. (2017). Effects of metal-rich particulate matter exposure on exogenous and endogenous viral sequence methylation in healthy steel-workers. *Environ. Res.* 159 452–457. 10.1016/j.envres.2017.08.042 28858759

[B52] Meyer T. J. Rosenkrantz J. L. Carbone L. Chavez S. L. (2017). Endogenous retroviruses: with us and against us. *Front. Chem.* 5:23 10.3389/fchem.2017.00023 PMC5384584 28439515

[B53] Ohnuki M. Tanabe K. Sutou K. Teramoto I. Sawamura Y. Narita M. (2014). Dynamic regulation of human endogenous retroviruses mediates factor-induced reprogramming and differentiation potential. *Proc. Natl. Acad. Sci. U.S.A.* 111 12426–12431. 10.1073/pnas.1413299111 25097266 PMC4151758

[B54] Paoli P. Giannoni E. Chiarugi P. (2013). Anoikis molecular pathways and its role in cancer progression. *Biochim. Biophys. Acta* 1833 3481–3498. 10.1016/j.bbamcr.2013.06.026 23830918

[B55] Pérot P. Mullins C. S. Naville M. Bressan C. Hühns M. Gock M. (2015). Expression of young HERV-H loci in the course of colorectal carcinoma and correlation with molecular subtypes. *Oncotarget* 6 40095–40111. 10.18632/oncotarget.5539 26517682 PMC4741882

[B56] Plaks V. Kong N. Werb Z. (2015). The cancer stem cell niche: how essential is the niche in regulating stemness of tumor cells? *Cell Stem Cell* 16 225–238. 10.1016/j.stem.2015.02.015 25748930 PMC4355577

[B57] Ramos E. K. Hoffmann A. D. Gerson S. L. Liu H. (2017). new opportunities and challenges to defeat cancer stem cells. *Trends Cancer* 3 780–796. 10.1016/j.trecan.2017.08.007 29120754 PMC5958547

[B58] Reis B. S. Jungbluth A. A. Frosina D. Holz M. Ritter E. Nakayama E. (2013). Prostate cancer progression correlates with increased humoral immune response to a human endogenous retrovirus GAG protein. *Clin. Cancer Res.* 19 6112–6125. 10.1158/1078-0432.CCR-12-3580 24081977

[B59] Renner K. Singer K. Koehl G. E. Geissler E. K. Peter K. Siska P. J. (2017). Metabolic Hallmarks of Tumor and Immune Cells in the Tumor Microenvironment. *Front. Immunol.* 8:248. 10.3389/fimmu.2017.00248 28337200 PMC5340776

[B60] Roesch A. (2015). Tumor heterogeneity and plasticity as elusive drivers for resistance to MAPK pathway inhibition in melanoma. *Oncogene* 34 2951–2957. 10.1038/onc.2014.249 25109330

[B61] Romanish M. T. Cohen C. J. Mager D. L. (2010). Potential mechanisms of endogenous retroviral-mediated genomic instability in human cancer. *Semin. Cancer Biol.* 20 246–253. 10.1016/j.semcancer.2010.05.005 20685251

[B62] Sandoval J. Esteller M. (2012). Cancer epigenomics: beyond genomics. *Curr. Opin. Genet. Dev.* 22 50–55. 10.1016/j.gde.2012.02.008 22402447

[B63] Schanab O. Humer J. Gleiss A. Mikula M. Sturlan S. Grunt S. (2011). Expression of human endogenous retrovirus K is stimulated by ultraviolet radiation in melanoma. *Pigment Cell Melanoma Res.* 24 656–665. 10.1111/j.1755-148X.2011.00860.x 21501418

[B64] Serafino A. Balestrieri E. Pierimarchi P. Matteucci C. Moroni G. Oricchio E. (2009). The activation of human endogenous retrovirus K (HERV-K) is implicated in melanoma cell malignant transformation. *Exp. Cell Res.* 315 849–862. 10.1016/j.yexcr.2008.12.023 19167380

[B65] Sinibaldi-Vallebona P. Matteucci C. Spadafora C. (2011). Retrotransposon-encoded reverse transcriptase in the genesis, progression and cellular plasticity of human cancer. *Cancers* 3 1141–1157. 10.3390/cancers3011141 24212657 PMC3756407

[B66] Skvortsov S. Debbage P. Cho W. C. Lukas P. Skvortsova I. (2014). Putative biomarkers and therapeutic targets associated with radiation resistance. *Expert Rev. Proteomics* 11 207–214. 10.1586/14789450.2014.893194 24564737

[B67] Stengel S. Fiebig U. Kurth R. Denner J. (2010). Regulation of human endogenous retrovirus-K expression in melanomas by CpG methylation. *Genes Chromosomes Cancer* 49 401–411. 10.1002/gcc.20751 20095041

[B68] Subramanian R. P. Wildschutte J. H. Russo C. Coffin J. M. (2011). Identification, characterization, and comparative genomic distribution of the HERV-K (HML-2) group of human endogenous retroviruses. *Retrovirology* 8:90. 10.1186/1742-4690-8-90 22067224 PMC3228705

[B69] Suntsova M. Garazha A. Ivanova A. Kaminsky D. Zhavoronkov A. Buzdin A. (2015). Molecular functions of human endogenous retroviruses in health and disease. *Cell Mol. Life Sci.* 72 3653–3675. 10.1007/s00018-015-1947-6 26082181 PMC11113533

[B70] Taddei M. L. Giannoni E. Comito G. Chiarugi P. (2013). Microenvironment and tumor cell plasticity: an easy way out. *Cancer Lett.* 341 80–96. 10.1016/j.canlet.2013.01.042 23376253

[B71] Toufaily C. Landry S. Leib-Mosch C. Rassart E. Barbeau B. (2011). Activation of LTRs from different human endogenous retrovirus (HERV) families by the HTLV-1 tax protein and T-cell activators. *Viruses* 3 2146–2159. 10.3390/v3112146 22163338 PMC3230845

[B72] van den Hurk K. Niessen H. E. Veeck J. van den Oord J. J. van Steensel M. A. Zur Hausen A. (2012). Genetics and epigenetics of cutaneous malignant melanoma: a concert out of tune. *Biochim. Biophys. Acta* 1826 89–102. 10.1016/j.bbcan.2012.03.011 22503822

[B73] Vargas A. Moreau J. Landry S. LeBellego F. Toufaily C. Rassart E. (2009). Syncytin-2 plays an important role in the fusion of human trophoblast cells. *J. Mol. Biol.* 392 301–318. 10.1016/j.jmb.2009.07.025 19616006

[B74] Wang J. Xie G. Singh M. Ghanbarian A. T. Raskó T. Szvetnik A. (2014). Primate-specific endogenous retrovirus-driven transcription defines naive-like stem cells. *Nature* 516 405–409. 10.1038/nature13804 25317556

[B75] Wang M. Zhao J. Zhang L. Wei F. Lian Y. Wu Y. (2017). Role of tumor microenvironment in tumorigenesis. *J. Cancer* 8 761–773. 10.7150/jca.17648 28382138 PMC5381164

[B76] Wang-Johanning F. Rycaj K. Plummer J. B. Li M. Yin B. Frerich K. (2012). Immunotherapeutic potential of anti-human endogenous retrovirus-K envelope protein antibodies in targeting breast tumors. *J. Natl. Cancer Inst.* 104 189–210. 10.1093/jnci/djr540 22247020 PMC3274512

[B77] White C. H. Beliakova-Bethell N. Lada S. M. Breen M. S. Hurst T. P. Spina C. A. (2018). Transcriptional modulation of human endogenous retroviruses in primary CD4+ T cells following vorinostat treatment. *Front. Immunol.* 12:603. 10.3389/fimmu.2018.00603 29706951 PMC5906534

[B78] Wildschutte J. H. Williams Z. H. Montesion M. Subramanian R. P. Kidd J. M. Coffin J. M. (2016). Discovery of unfixed endogenous retrovirus insertions in diverse human populations. *Proc. Natl. Acad. Sci. U.S.A.* 113 E2326–E2334. 10.1073/pnas.1602336113 27001843 PMC4843416

[B79] Yamasaki S. Taguchi Y. Shimamoto A. Mukasa H. Tahara H. Okamoto T. (2014). Generation of human induced pluripotent stem (Ips) cells in serum- and feeder-free defined culture and TGF-B1 regulation of pluripotency. *PLoS One* 9:e87151. 10.1371/journal.pone.0087151 24489856 PMC3906124

[B80] Young G. R. Stoye J. P. Kassiotis G. (2013). Are human endogenous retroviruses pathogenic? An approach to testing the hypothesis. *Bioessays* 35 794–803. 10.1002/bies.201300049 23864388 PMC4352332

[B81] Yun J. Johnson J. L. Hanigan C. L. Locasale J. W. (2012). Interactions between epigenetics and metabolism in cancers. *Front. Oncol.* 15:163. 10.3389/fonc.2012.00163 23162793 PMC3498627

[B82] Zhu Y. Luo M. Brooks M. Clouthier S. G. Wicha M. S. (2014). Biological and clinical significance of cancer stem cell plasticity. *Clin. Transl. Med.* 3:32. 10.1186/s40169-014-0032-3 26932376 PMC4883980

